# Retraction of ‘Dissecting the splicing mechanism of the *Drosophila* editing enzyme; *dADAR*’

**DOI:** 10.1093/nar/gkae221

**Published:** 2024-04-11

This is a retraction of: Roberto Marcucci, Maurizio Romano, Fabian Feiguin, Mary A. O'Connell, Francisco E. Baralle, Dissecting the splicing mechanism of the Drosophila editing enzyme; dADAR, *Nucleic Acids Research*, Volume 37, Issue 5, 1 April 2009, Pages 1663–1671, https://doi.org/10.1093/nar/gkn1080

Following allegations of image manipulation in Figure 3C in 2023, the journal conducted a brief investigation and detected potential issues in other figures.

1. Figure 3C: lanes 1 and 2 (Testis and Ovaries) appear to be duplicates.
2. Figure 3C, RP49, lanes 5–6, 7–8 and Figure 4B, Egl control, lanes 1–2 appear identical after size adjustments.
3. Column charts in Figures 1, 3, 4: the error bars appear to have been added by hand.

The matter was referred to the authors’ institution and an Expression of Concern was published.

The authors have since been unable to produce the original data. The institutional investigation substantiated the issues raised about Figures 3C, lanes 1 and 2, and concluded that the lanes are duplicates.

The institutional investigation concluded the following about the other images:

- Figure 3C, RP49, lanes 5–6, 7–8 and Figure 4B, Egl control, lanes 1–2: close examination of the images indicate that these lanes are not direct copies of each other. Bands are only similar after digital manipulation such as cropping and resizing.
- Column charts in Figures 1, 3, 4: the error bars have likely been added by hand.

Further examination by the Editors reveals that, in addition to the error bars, some columns also appear to have been added by hand to the column graphs.

Based on their assessment of the institutional investigation and the figures, the Editors have determined that the image manipulations described above undermine the credibility of the study and have decided to retract the article. The authors have been informed of this decision.

